# Supplementary material for: Planetary-scale streak structure reproduced in high-resolution simulations of the Venus atmosphere with a low-stability layer
Source: Nat Commun. 2019 Jan 9;10:23. doi: 10.1038/s41467-018-07919-y (PMC6327047; doi:10.1038/s41467-018-07919-y)
Supplement: Supplementary file 1 — Supplementary Information [file 41467_2018_7919_MOESM1_ESM.pdf]

Supplementary Figure of

Planetary-scale streak structure reproduced in high-resolution simulations of the Venus atmosphere with a low-stability layer

Kashimura *et al*.

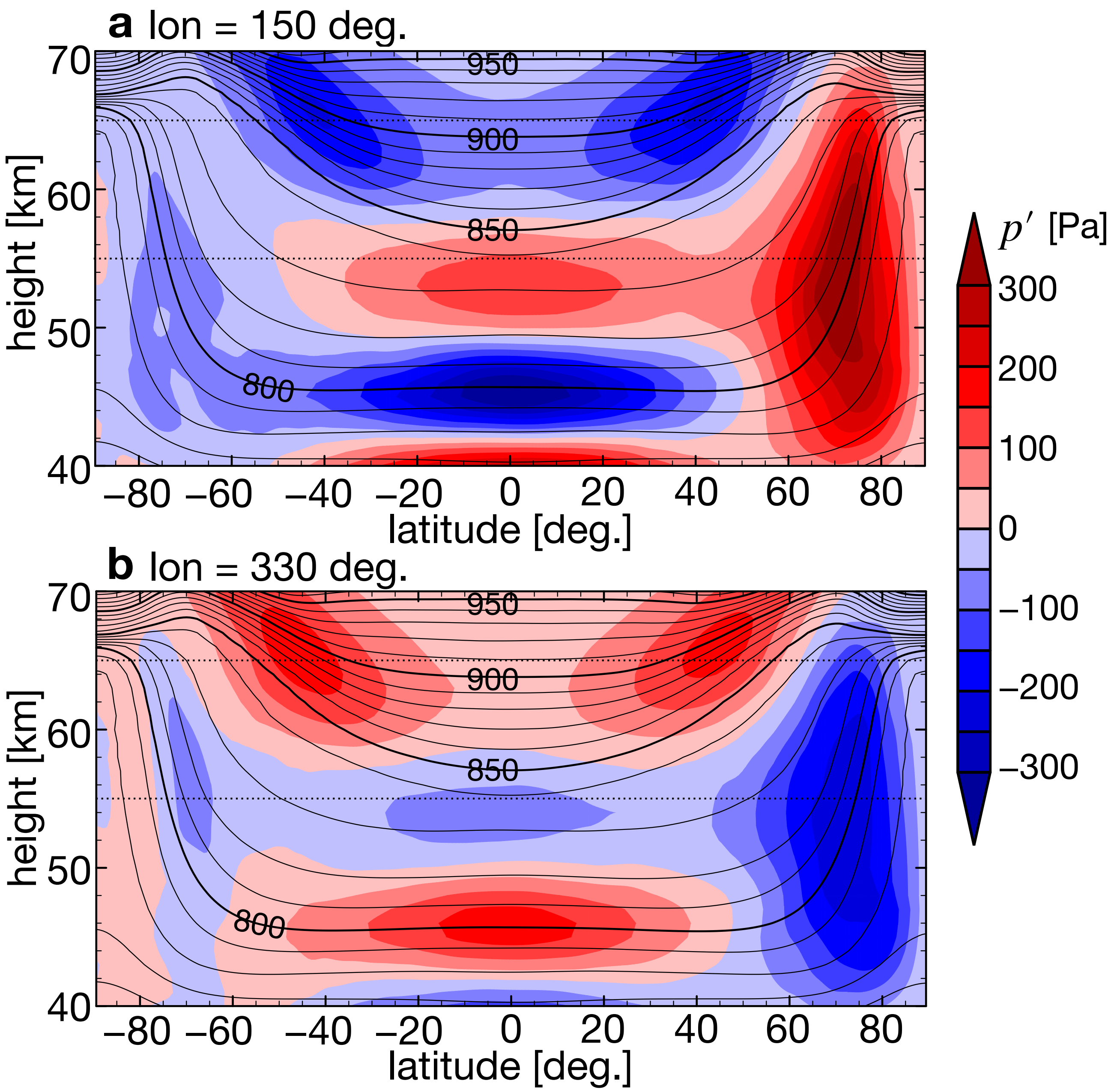

Supplementary Figure 1: Latitude-height cross-sections of the pressure anomaly (colour) in the composite mean of the ZS0 case. Longitudes are (a) 150 deg. and (b) 330 deg. Contours indicate the zonally and temporally averaged potential temperature that is the same as Fig. 5f. The contour interval is 10 K. Horizontal dotted lines show heights of 55 km and 65 km.
