# Supplementary material for: Planetary-scale streak structure reproduced in high-resolution simulations of the Venus atmosphere with a low-stability layer
Source: Nat Commun. 2019 Jan 9;10:23. doi: 10.1038/s41467-018-07919-y (PMC6327047; doi:10.1038/s41467-018-07919-y)
Supplement: Supplementary file 3 — Description of Additional Supplementary Files [file 41467_2018_7919_MOESM3_ESM.pdf]

Supplementary Movie 1: Polar views of vertical velocity at 60 km height in the STD case after 3 Earth years of time-integration. Data output interval is 24 hours. Left and right circles show the southern and northern hemispheres, respectively. Outermost circles are the equator and dashed circles are 30-deg. and 60-deg. latitude circles. Small red arcs indicate the sub-solar point.

Supplementary Movie 2: Same as Supplementary Movie 1 but for after 4 Earth years of time-integrations and with a data output interval of 1 hour.

Supplementary Movie 3: Same as Supplementary Movie 1 but for the ZS0 case, in which the solar heating is zonally symmetric. Data output interval is 6 hours.
